# Transcorneal aspiration for management of primary iris cysts in the standing horse

**DOI:** 10.1002/vms3.1570

**Published:** 2024-07-30

**Authors:** Stefanie Conduit, Mark Bowen, Gayle Hallowell, Regina Pereira, Guilia Rapezzano, Adam Redpath

**Affiliations:** ^1^ School of Veterinary Medicine and Science University of Nottingham Sutton Bonington UK; ^2^ Equine Medicine Veterinary Referrals Melton Mowbray UK; ^3^ Pool House Equine Hospital, Part of IVC Evidensia Fradley UK; ^4^ Donnington Grove Equine Hospital, Part of IVC Evidensia Newbury UK

**Keywords:** copra nigra, equine, iris cyst, ophthalmology, transcorneal aspiration

## Abstract

**Background:**

Equine primary iris cysts are usually incidental findings but, if associated with clinical signs, may require intervention. The use of laser (Nd:Yag or diode) has been reported but requires specialised equipment. Transcorneal aspiration has not been previously evaluated in the standing horse.

**Objectives:**

To review outcomes of standing transcorneal aspiration of primary iris cysts (STAPIC) in horses.

**Methods:**

Horses were identified from electronic patient records from 2018 to 2024 across four collaborating centres. Clinical presentation and outcomes were identified and reported using descriptive statistics.

**Results:**

Eighteen horses were identified. Behavioural signs reported included ‘spooking’ and changes in rideability often associated with jumping. Single large unilateral cysts were present in 11 horses, bilateral cysts in three horses and multiple unilateral cysts in four horses. Following treatment, one horse developed uveitis and fibrin in the anterior chamber associated with needle contact with the iris stroma due to movement, and a second horse developed fibrin within the anterior chamber. Both conditions resolved with anti‐inflammatory medication and administration of tissue plasminogen activator. No other adverse effects were reported. Follow‐up was available from all horses (median: 6 months, interquartile range [IQR]: 4–11 months) with no recurrence, although one horse developed an iris cyst in the contralateral eye after 3 years. All owners reported improvement in clinical signs, with 61% reporting no further signs.

**Conclusions:**

STAPIC is an effective and easily accessible alternative for treating iris cysts in horses rarely associated with complications.

## Graphical abstract

Primary iris cysts, although incidental, can cause clinical signs associated with behavioural change and therefore may require treatment. Where treatment is required, cyst deflation either using a laser or the STAPIC technique is recommended. STAPIC is an effective and easily accessible alternative for treating iris cysts in horses and resulted in few complications, which were manageable without long‐term consequences.

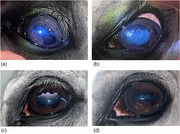

## 1 INTRODUCTION

Equine primary iris cysts are smooth, often focal swellings that are darker than the iris and usually attached to the pupillary margin, arising from the inner non‐pigmented ciliary body epithelium (Dziezyc et al., 1990; Shields et al., 1984). They are often an incidental finding in the horse but have been reported to cause behavioural changes including spooking, poor performance often associated with jumping and headshaking (Berger et al., 2008; Stas et al., 2023). Occasionally, they have been reported to contact the corneal endothelium, leading to oedema formation with recurrent corneal ulceration (Byam‐Cook & Knottenbelt, 2007; Stas et al., 2023). Theoretically, they can also affect aqueous humour flow, although secondary glaucoma has not been reported in horses (Gemensky‐Metzler et al., 2004; Gilger et al., 1997; Stas et al., 2023).

In the horse, iris cysts are most commonly found dorsally, often associated with the corpora nigra (Gilger et al., 2022), although they can be found at other locations within the eye including the ciliary body, iris stroma and anterior chamber. The cyst is usually tethered to the pupillary margin and is rarely free‐floating, in contrast to dogs (Berger et al., 2008; Gemensky‐Metzler et al., 2004). Cysts may be congenital and have been observed in very young horses; however, many older horses can develop iris cysts spontaneously (Gilger et al., 2022). They can be present unilaterally or bilaterally and can be present as multiple cysts within one eye. Cyst size can vary from very small to moderate to large and can interfere with vision, which is reported to be accentuated in bright light due to pupillary miosis (Gilger et al., 2022).

Treatment is recommended if the cyst is suspected to be associated with clinical signs. Multiple studies have reported improvements in behaviour including spooking and headshaking following cyst deflation (Gemensky‐Metzler et al., 2004; Lam & Pumphrey, 2021). Horses with bilateral cysts have been reported to demonstrate behavioural problems more frequently than those with unilateral cysts (Lam & Pumphrey, 2021). The current recommended treatment is transcorneal ablation to deflate the cyst using a diode, argon or neodymium–yttrium aluminium garnet (Nd:YAG) laser utilising standing sedation. Multiple case series have reported successful treatment with minimal complications (Gemensky‐Metzler et al., 2004; Lam & Pumphrey, 2021; Stas et al., 2023; Tóth & Buijs, 2018). Successful treatment of iris cysts using transcorneal fine needle aspiration has been reported under general anaesthesia in a range of species (Byam‐Cook & Knottenbelt, 2007; Delgado et al., 2010; Georgalas et al., 2018). However, this has not been reported using standing sedation on the horse.

This study aimed to evaluate the outcome of standing transcorneal aspiration of primary iris cysts (STAPIC) across four collaborating centres in addition to determining the most frequent complications. We hypothesised that STAPIC is an effective method for primary iris cyst reduction with a low complication rate.

## 2 MATERIALS AND METHODS

### 2.1 Cases

Horses that underwent treatment for primary iris cysts using the STAPIC technique were identified from the electronic patient records across four collaborating centres from 2018 to 2024. The presenting clinical signs and location and number of cysts on presentation were extracted from the records alongside previous diagnostics, reported findings of transcorneal ultrasound, treatment and outcomes. Inclusion criteria were that horses had had a full ophthalmic examination performed by an ECEIM or ACVIM diplomate which included slit lamp biomicroscopy, trans‐illumination and direct ophthalmoscopy. Horses must have undergone the procedure at one of the four collaborating centres. Horses were excluded if follow‐up was not performed or adequately recorded to enable assessment of outcome. Where ocular ultrasound was performed, the findings were recorded. All ultrasound examinations were undertaken using a standard transpalpebral approach using a linear high‐frequency probe (at least 12 MHz) to confirm the identified structures were fluid filled, confirming the diagnosis as an iris cyst. The location and number of cysts were recorded, along with any association with the corpora nigra.

### 2.2 Procedure

The standard approach included intravenous sedation with detomidine (typically 0.01 mg/kg, Domidine; Dechra Veterinary Products) and butorphanol (typically 0.01 mg/kg IV, Torphadine; Dechra Veterinary Products). Where animals were responsive to stimulation, further boluses were administered. Local anaesthesia was provided using perineural anaesthesia of the auriculopalpebral and frontal nerves with mepivacaine (40 mg; 20 mg/mL; 2 mL; Intra‐Epicaine; Dechra Veterinary Products) and topical anaesthesia using proxymetacaine hydrochloride 0.5% or tetracaine hydrochloride 0.5% (Bausch & Lomb). Mydriasis was performed using topical tropicamide 1% (Bausch & Lomb) or atropine sulphate 1% (Bausch & Lomb). Pre‐operative analgesia was provided with intravenous flunixin meglumine (1.1 mg/kg, Finadyne; MSD Animal Health). Aseptic preparation, when undertaken, involved the use of 5% povidone‐iodine applied to the cornea and cleansing of the dorsal and ventral fornix using a swab.

The procedure was performed using a narrow‐gauge needle or cannula stylet (27‐G 1.5″ needle/25‐G 2″ cannula stylet) introduced through the cornea, using a transcorneal approach, close to the limbus. The entry point was determined by cyst location, with the aim to enter the cornea as close to the cyst as possible. The cyst was perforated and aspirated using an extension set and syringe, often with an assistant to aid aspiration. Additionally, the cyst was perforated on the far side by passing the needle completely through the cyst in most cases. If multiple cysts were present, the needle was redirected within the aqueous chamber to avoid multiple entries into the cornea where possible.

Post‐operative management was clinician dependent. Some cases received topical prednisolone twice daily for 2 days (0.2 mL/eye; Pred Forte) and oral phenylbutazone twice daily for 2 days (2.2 mg/kg; Equipalazone; Dechra Veterinary Products); others only received pre‐operative flunixin and no further medications. Management recommendations given included box rest for 3–5 days and application of a fly mask until the normal pupillary response returned.

### 2.3 Follow‐up

Horses were re‐examined 24 h following the procedure in person and then in person or via a photograph at 6 weeks. Long‐term follow‐up was performed either in person if the horse represented to the hospital or by phone call and photograph at variable time intervals. The in‐person assessment at 24 h and at 6 weeks included assessing cyst deflation and a full ophthalmic exam to assess for any changes to the eye, particularly the cornea and lens. The photographs were evaluated for effective cyst deflation and for assessing the cornea and anterior chamber as far as possible. Both the in‐person and photographic assessments also included the following questions outlined as part of the phone conversations: *Have you noticed any changes in your horse since the procedure?* and where clinical signs were reported, *Have the clinical signs you reported changed since the procedure and would you classify these as no change, mild improvement, marked improvement or worsening?*

All outcomes and statistics reported are descriptive. Age and follow‐up were not normally distributed and so are reported as median (interquartile range [IQR]).

## 3 RESULTS

Eighteen horses were identified with a total of 20 affected eyes. Median age at presentation was 11 years (IQR: 9–14 years), and the population included 13 geldings and five mares. There was a wide range of breeds including Thoroughbred (*n* = 4), Irish Sport Horse (*n* = 6), Cob (*n* = 3), Warmblood (*n* = 2), Connemara (*n* = 2) and Irish Draught (*n* = 1). Clinical signs reported at presentation included spooking (10/18), changes in rideability (7/18), reduced vision in dark areas (2/18), anorexia at pasture in the summer (1/18) and head shaking (1/18). Additionally, two horses were presented following cyst detection at a pre‐purchase examination, leading to a request for treatment prior to the horse being sold. One of these horses was reported to have changes in rideability associated with dark corners; the other had no appreciable clinical signs. In all cases, other common differential diagnoses for the clinical signs reported were excluded using diagnostics. The diagnostics used varied depending on the presenting clinical sign.

Eleven horses had unilateral single cysts, four horses had unilateral cysts with multiple present within one eye (polycystic) and three horses had bilateral cysts, of which one was polycystic and the other two had single cysts in both eyes. Cysts were present in the left eye in seven horses, the right eye in eight horses and bilaterally in three horses. Ocular ultrasound was performed in 83% of horses (15/18). All cysts were fluid filled. In four cases, the cysts were associated with the corpora nigra of which one was polycystic. All other cysts were isolated cysts located medially, ventrally or laterally.

**FIGURE 1:**
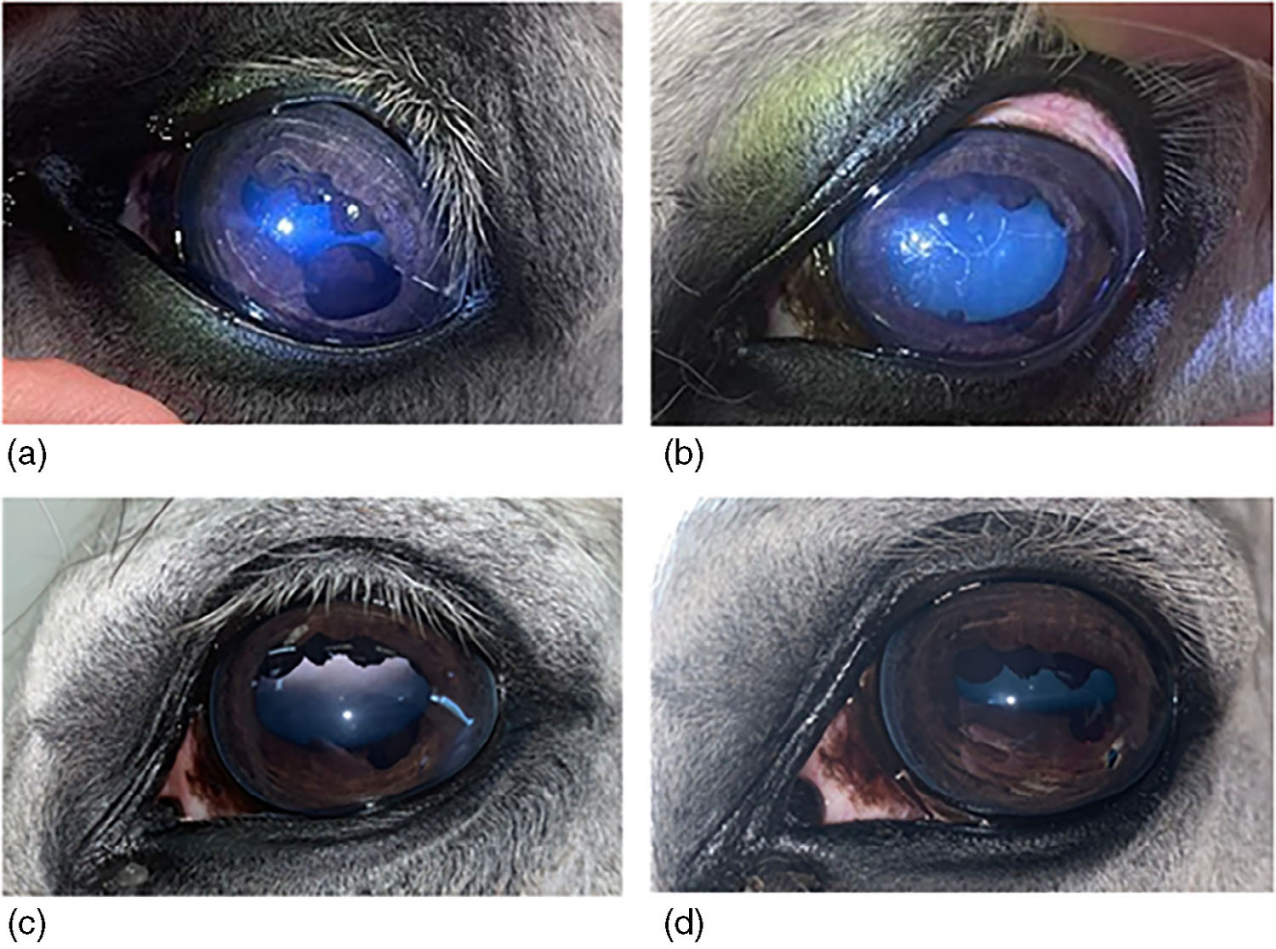
Images showing iris cyst prior to treatment (a), immediately following treatment with STAPIC (spider web appearance on cornea is due to refraction from the topical medications) (b), 24 h after treatment with STAPIC (c), and 6 weeks after treatment with STAPIC (d).

All horses underwent the procedure as described in the methods, and in all cases, the cysts were completely deflated (Figure 1, Video 1). Two horses received pre‐operative aseptic preparation, and the other cases were all performed with no aseptic preparation but with an aseptic technique (sterile gloves, sterile needle, syringe and extension set). Topical tropicamide 1% (Bausch & Lomb) or atropine sulphate 1% (Bausch & Lomb) was used to dilate the pupil in eight and ten horses, respectively. A 27‐G 1.5″ needle was used in five eyes, and a 25‐G 2″ cannula stylet was used in 13 eyes. Approximately 0.1 mL of cyst fluid was aspirated, although this varied with cyst size. All cysts were easy to puncture. Where multiple cysts were present, the needle was redirected to enable aspiration of all cysts in four cases; however, in one case, this was not possible, and the needle was introduced twice. This case had two cysts present dorsally associated with the corpora nigra, and therefore, excessive needle movement within the anterior chamber was avoided to reduce the risk of contacting the unaffected aspects of the corpora nigra.

**FIGURE 2:**
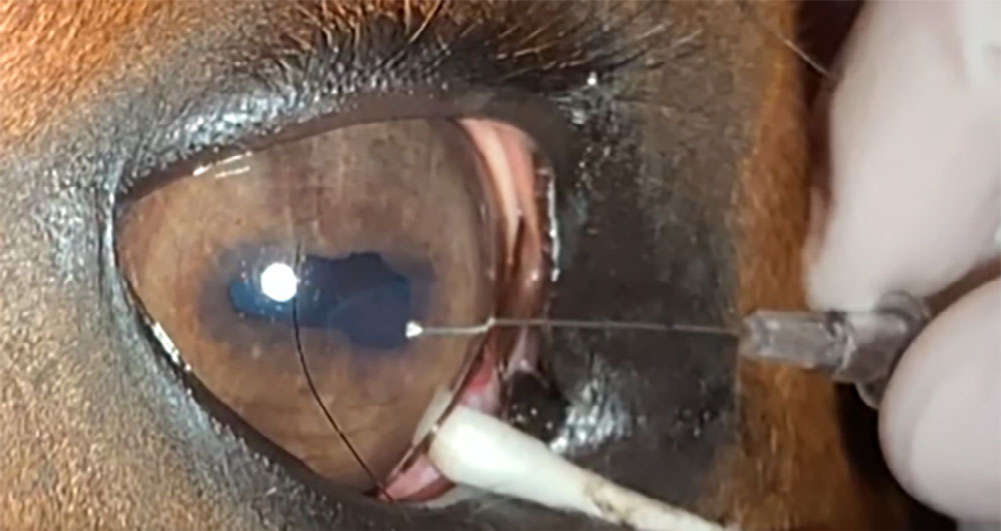
Image showing placement of needle to aspirate a medial iris cyst. Note the cotton bud holding the third eyelid in position.

Intraoperative complications included horse movement leading to iris contact with the needle (1/18), excessive third eyelid movement (2/18) and cyst material being aspirated into the cornea (2/18). Third eyelid movement was reduced by using a sterile cotton bud to prevent excessive movement (Figure 2). In both cases where cyst material herniated through the needle tract, it was immediately retracted back into the cornea by gentle pressure around the needle hole without any short‐ or long‐term complications.

Post‐operative complications and long‐term follow‐up are reported in Table 1. Median follow‐up time was 6 months (IQR: 4–11 months, range: 6 weeks to 3 years). At 6 weeks, all owners reported an improvement in clinical signs, with 61% reporting no further signs and 39% reporting a marked improvement. No owners reported a mild improvement or worsening of clinical signs. There was no cyst re‐occurrence, although one horse had developed a new iris cyst when re‐examined 3 years following treatment in the contralateral eye.

**TABLE 1:** Presenting clinical signs, number of cysts, complications observed, and follow‐up for each horse following treatment with STAPIC.

		Cysts present				Additional follow‐up		
	Clinical signs	Left eye	Right eye	Complications	6‐week follow‐up	When? (years)	How?	Outcome reported by owner
1	Spooking	0	1		In person	1	Photo	Improved
2	Changes in rideability	1	2		In person	2	Photo	Improved
3	Changes in rideability	1	0		In person	2	Photo	Resolved
4	Spooking	1	0		In person	2	Photo	Resolved
5	Spooking	2	0		In person	0.5	Photo	Resolved
6	Failed pre‐purchase exam Changes in rideability in dark corners (will not jump)	0	1		In person	0.5	Photo	Resolved
7	Spooking in dark corners	1	0		In person	0.3	Photo	Resolved
8	Changes in rideability (will not jump)	2	0		In person	0.12	In person	Resolved
9	Spooking	0	1	Fibrin in AC	Photo	0.12	Photo	Improved
10	Behavioural change and anorexia in the summer	1	1		Photo	0.5	In person	Resolved
11	Changes in rideability	2	0		In person	3	In person	Resolved. New cyst in right eye.
12	Spooking	0	1		In person	0.5	In person	Improved
13	Spooking	0	1	Uveitis and fibrin in AC	In person	0.5	In person	Improved
14	Head shaking and spooking	1	0		In person	0.5	In person	Resolved
15	Changes in rideability (will not jump)	0	1		In person	0.5	In person	Improved
16	Spooking	1	1		In person	0.5	In person	Improved
17	Changes in rideability (struggling to jump) and spooking	0	1		In person	0.12	In person	Resolved
18	Noticed at PPE	0	2		In person	0.12	In person	Resolved

Abbreviations: AC, anterior chamber; STAPIC, standing transcorneal aspiration of primary iris cysts.

Sixteen horses received topical prednisolone and oral phenylbutazone for 2 days, and two horses received no post‐operative medications. Neither of these horses developed any signs of ocular discomfort or uveitis despite not receiving post‐operative anti‐inflammatories.

Post‐operative complications occurred in two horses. One horse developed anterior chamber fibrin deposition without other signs of uveitis, and the other developed fibrin deposition with clinical signs of uveitis including ptosis, blepharospasm, epiphora and miosis. These were both observed within 24 h of the procedure. The horse that developed the uveitis was the same horse that had iris contact with the needle during the procedure; therefore, it is suspected this was the cause of uveitis development. Both horses were treated with tissue plasminogen activator (TPA) injection into the anterior chamber of the eye in addition to the standard post‐operative plan and atropine to effect. The horse with uveitis and fibrin deposition responded to treatment within 48 h, with no further complications noted. The horse with only fibrin deposition initially received TPA injection into the anterior chamber twice 24 h apart followed by topical prednisolone therapy for 2 weeks until the fibrin resolved. There was no evidence of corneal scarring, corneal ulceration, cataracts or infection noted at any follow‐up evaluation.

## 4 DISCUSSION

This study describes a transcorneal technique to deflate primary iris cysts in the horse with minimal complications. To date, there have been no other published reports of this technique being used in a standing sedated horse. A similar technique has been described in the horse using a translimbal approach with a 27‐ to 30‐G needle attached to a tuberculin syringe (Gilger et al., 2022). It is not stated whether this procedure was done under standing sedation or general anaesthesia. Additionally, the chapter describes the procedure as invasive with postoperative treatment using topical anti‐inflammatory and mydriatic medications for the resultant uveitis, although does not provide any references to demonstrate evidence for this statement. A case report in the horse used a 25‐G needle under general anaesthesia to perform aspiration using a translimbal approach with complete regression of the iris cyst leaving a stalk remnant attached to the pupillary margin with no complications from the aspiration (Byam‐Cook & Knottenbelt, 2007). In other species, this technique has also been reported under general anaesthesia, although they do not outline if the approach was transcorneal or translimbal (Delgado et al., 2010; Georgalas et al., 2018).

Prior to treatment, primary iris cysts were differentiated from other differential diagnoses including intraocular melanoma and corpora nigra hyperplasia using ultrasound in most cases. Ultrasound can be performed using a transpalpebral technique with a linear probe (Hallowell & Bowen, 2007), and iris cysts are typically rounded structures with a thin hyperechoic edge surrounding an anechoic centre (Whitcomb, 2002). Ultrasound is reported to rarely be required as iris cysts have a very characteristic appearance (Gilger et al., 2022); however, Stas et al. (2023) reported that ultrasound prior to treatment aided in evaluating the number of cysts and their appearance including the size, architecture and wall thickness. They found this to be useful information to determine treatment success rates and surgical planning when using a diode laser. This was particularly important for thick‐walled cysts and polycystic eyes. In this study, cyst wall thickness was not assessed; however, we observed no difference in treatment outcome with polycystic eyes. Ultrasound in this study did not add to treatment planning but confirmed the presence of the cyst. When using a diode laser, the wall thickness of the cyst is clinically relevant in terms of determining the amount of energy required. This consideration is less relevant to needle aspiration. Cysts were not commonly associated with corpora nigra in this population of horses, unlike previous descriptions (Gilger et al, 2022), although the significance of this is uncertain.

Complications in this study were rare and were associated with the movement of the horse, the third eyelid or the globe. To try to reduce horse movement, adequate sedation is essential to minimise intraoperative complications. Adequate sedation was deemed to be achieved when the horse was non‐responsive to external stimuli. Temperament must be considered as fractious horses may not be amenable to this as a standing procedure even with adequate sedation. All procedures were performed in a hospital environment, using stocks and a headstand to help reduce the risk of horse movement. Third eyelid movement was reduced using a sterile cotton bud; however, this does not affect globe movement. A retrobulbar nerve block could be performed which would prevent globe movement (McKinney, 2021). Our initial expectations were that a retrobulbar block might not be necessary, but from our experience, it would have been beneficial in two of 18 cases. Given the limited number of horses in this study, further investigation with a larger sample size would provide a more definitive conclusion regarding the necessity of a retrobulbar block. Additionally, this technique was performed via a transcorneal approach rather than a translimbal approach to enable easier passage of the needle in the standing horse.

This procedure is only feasible with a stationary cyst in a standing horse; therefore, if the cyst is free‐floating, this procedure is not recommended. In horses, the cyst is usually tethered to the pupillary margin in contrast to dogs where free‐floating cysts are more common (Berger et al., 2008; Gemensky‐Metzler et al., 2004).

In this study, most horses were presented due to owner concerns with behaviour or performance. Clinical signs of primary iris cysts are not pathognomonic, and therefore, other potential causes of the presenting clinical sign were excluded prior to the decision to treat the iris cyst. It is impossible to know prior to treatment whether the cyst is responsible for the clinical signs as there is no confirmatory diagnostic test. However, examining the pupil size without pharmacological dilation in bright light can allow for an assessment when the pupil is miotic, allowing for an estimation of pupillary aperture blockage caused by the iris cyst (Gilger et al., 2022). In this study, the significance of the lesion was ultimately confirmed by response to treatment as in previous studies (Berger et al., 2008; Gemensky‐Metzler et al., 2004; Lam & Pumphrey, 2021; Stas et al., 2023).

The most common clinical signs observed in this study included spooking and changes in rideability which is consistent with other published reports (Berger et al., 2008; Stas et al., 2023). However, two owners reported reduced vision in darker areas associated with spooking, where previously clinical signs have been reported to be accentuated in bright light due to miosis (Gilger et al., 2022). This could be due to the pupil size changing when moving from light to dark areas, potentially causing the cyst to move or making it more obvious to the horse.

Following treatment, all owners reported an improvement in clinical signs which is similar to previous studies (Gemensky‐Metzler et al., 2004; Lam & Pumphrey, 2021). There was no re‐occurrence of iris cysts comparable to most reports of treatment with laser ablation (Gemensky‐Metzler et al., 2004; Lam & Pumphrey, 2021; Tóth & Buijs, 2018); however, Stas et al. (2023) reported a re‐occurrence in one horse 11 months after the initial treatment with a diode laser. The outcomes of the study cannot be directly compared to those which used a laser; however, in the case series reported by Stas et al. (2023), 78% of treated eyes had a good decrease in iris cyst size, 15% had a moderate decrease in size and 4% showed minimal to no change in cyst size. In comparison, all the cysts treated in this study were completely deflated using STAPIC. In the report by Stas et al. (2023), those that only had a minimal to moderate effect were polycystic and hyperplastic corpora nigra. They report that when considering their cases, each additional cyst reduced the chance of treatment being successful. In this study, there were five horses which presented with multiple cysts within one eye, one of which was associated with the corpora nigra, and all the cysts were successfully treated using STAPIC, with all cysts being completely deflated. This suggests that when multiple cysts are present within one eye, STAPIC may provide a better treatment outcome; however, direct comparisons in a larger prospective study are needed to confirm this. Additionally, it has been reported that some iris cysts are difficult to deflate with significantly lower success reported with a laser due to the cyst being thick walled or lacking in pigment (Brooks, 2007; Stas et al., 2023). In this study, iris cyst wall thickness was not recorded, and no unpigmented cysts were observed. However, STAPIC should be an effective treatment for these iris cysts as it does not rely on the absorption of laser energy by the cyst but would require a direct comparison in a larger study with appropriate cases to confirm.

There have been no comparative studies to show that laser ablation is safer than STAPIC. Laser ablation has been associated with a number of complications including corneal oedema secondary to thermal endothelial damage, haemorrhage within the anterior chamber, anterior uveitis, corneal and anterior lens capsule pigmentation and retinal burns (Stas et al., 2023; Gemensky‐Metzler et al., 2004; Gilger et al., 1997). Although these have not been observed in a recent case series in horses of laser ablation, that study relied on non‐specialist follow‐up that may have missed subtle changes (Lam & Pumphrey, 2021). It is important to remember that any intraocular procedure can result in complications including inflammation (McColgin & Heier, 2000) and thus all horses should be closely monitored for complications following the procedure. All complications observed in this study can be attributed to intraocular inflammation and they occurred at a similar rate to those reported with laser ablation in the studies reported by Gemensky‐Metzler et al. (2004) and Stas et al. (2023). Gemensky‐Metzler et al. (2004) used a semiconductor diode laser in nine horses with follow‐up available in eight cases. They provided a peri‐operative dose of intravenous flunixin meglumine and topical atropine at the time of surgery and did not provide further post‐operative anti‐inflammatory therapy unless clinical signs of intraocular inflammation were observed. Two horses represented with mild blepharospasm 24 h later, which was controlled with a single dose of flunixin meglumine. Stas et al. (2023) reported a case series of 35 horses with iris cysts treated with a diode laser. Following the procedure, they observed damage to the retina in one case, localised corneal oedema leading to mild corneal fibrosis in three cases and uveitis and fibrin deposition in the anterior chamber in one case. They used a tapering dose of topical dexamethasone disodium phosphate 0.1% eyedrops for a variable time (3–24 days), and 29 of 35 horses received anti‐inflammatory therapy 1 day prior to the procedure in addition to meloxicam at the time of surgery. Other studies using laser ablation report no evidence of post‐operative uveitis (Gilger et al., 1997; Lam & Pumphrey, 2021; Tóth & Buijs, 2018). Tóth and Buijs (2018) and Lam and Pumphrey (2021) used both topical and systemic anti‐inflammatory therapy following the procedure. In comparison, Gilger et al. (1997) treated only four out of eight cases with topical anti‐inflammatory medication with no apparent difference between the treated and untreated groups in the development of post‐operative intraocular inflammation. This aligns with our experiences as two horses in this study received no post‐operative medications and did not develop any complications. Therefore, it is unclear whether post‐operative anti‐inflammatory medication is essential to prevent intraocular inflammation in all patients. A larger prospective study is required to investigate this further. Regardless of technique, it is essential to reduce trauma to other intraocular structures and subsequent inflammation, which requires minimal horse and globe movement. As outlined previously, a retrobulbar nerve block could be considered to minimise ocular movement. This warrants further investigation in a larger study to determine the need for a retrobulbar nerve block.

Pre‐operative antisepsis with povidone‐iodine was rarely performed in this study. For ocular surgery, there is evidence to support its use to reduce bacterial contamination and the risk of post‐operative endophthalmitis (Carrim et al., 2009; Speaker & Menikoff, 1991). For intraocular injections, although many studies advocate its use (Fischer et al., 2019; Launois et al., 2019), there are no studies which compare pre‐operative anti‐sepsis with povidone‐iodine with no antisepsis and the risk of developing post‐injection endophthalmitis. Additionally, a large human study has reported no complications following anterior chamber paracentesis with no pre‐operative antisepsis (Van der Lelij & Rothova, 1997). When considering ocular surgery, multiple studies have shown no difference in positive bacterial cultures when using povidone‐iodine compared to a placebo for cataract surgery (Soto & Mendivil, 2001; Walters et al., 1993). The majority of bacterial contamination in the eye originates from the eyelid, periorbital skin and conjunctiva (Leong et al., 2002). Unlike in ocular surgery, where contact with these tissues is unavoidable, a transcorneal injection into the anterior chamber has minimal risk of contamination from these tissues. Additionally, there is conflicting evidence regarding the concentration of povidone‐iodine required and its method of application, as its bactericidal activity is dependent on the concentration and exposure time (Hosseini et al., 2012). Concentrations of 5% have been found to be significantly more effective in reducing conjunctival bacterial flora than 1% (Ferguson et al., 2003). Currently, multiple guidelines recommend the application of 5% povidone‐iodine prior to cataract surgery with a 3‐min contact time (Koerner et al., 2018). However, corneal epithelial cell toxicity has been shown to increase with both povidone‐iodine exposure and concentration (Jiang et al., 2009; Shibata et al., 2014), and concentrations of greater than 0.1% are toxic to corneal endothelial cells (Naor et al., 2001). Recent studies advocate the use of repetitive dilute povidone‐iodine with a concentration of 0.25%, although this still does not provide complete sterilisation of the eye (Koerner et al., 2018). Povidone‐iodine has also been reported to cause contact or irritant dermatitis (Vandergriff et al., 2006). Due to the lack of evidence suggesting its requirement for intraocular injections, the difficulty of effective application to a standing sedated horse and the potential adverse effects, we often chose not to use a povidone‐iodine preparation prior to injection in this technique. We saw no evidence of infection in any case which did not receive an aseptic preparation following this procedure, suggesting that the risk of bacterial contamination following an intraocular injection is minimal. However, this should be assessed in a larger prospective study. A previous study of laser ablation of iris cysts used post‐procedural topical antibiotics (Lam & Pumphrey, 2021). No antibiotics were considered necessary in the cases identified in this case series.

Limitations of this study include that it is retrospective in nature, and therefore, there were some differences in how the cases were managed, and data collection relied on accurate record‐keeping. Although follow‐up was initially standardised at 24 h and 6 weeks, further follow‐up was performed at variable intervals and only via photographs in many cases. Additionally, follow‐up in clinical signs was performed by questioning owners rather than re‐examination. Not all cases had ocular ultrasonography prior to treatment, and intraocular pressures were not measured in any horse as there was no suspicion of glaucoma in any case.

## 5 CONCLUSION

Primary iris cysts, although incidental, can cause clinical signs associated with behavioural change and therefore may require treatment. Where treatment is required, cyst deflation either using a laser or the STAPIC technique is recommended. With either method, there is a risk of intraocular inflammation; therefore, close monitoring in addition to anti‐inflammatory therapy following the procedure is required. Effective sedation is essential to prevent complications associated with damage to the iris. Based on our experience, STAPIC appears to be a safe and effective procedure with minimal complications and does not require specialised equipment. A prospective comparison between laser and needle ablation of iris cysts is required to document whether either technique is superior. Until such data are presented, both techniques can be considered on a case‐by‐case basis.

## AUTHOR CONTRIBUTIONS

**Stefanie Conduit**: Data curation; formal analysis; investigation; writing—original draft; writing—review and editing. **Mark Bowen**: Conceptualisation; data curation; formal analysis; investigation; methodology; supervision; writing—review and editing. **Gayle Hallowell**: Conceptualisation; data curation; formal analysis; investigation; methodology; supervision; writing—review and editing. **Regina Pereira**: Investigation. **Giulia Rapezzano**: Investigation. **Adam Redpath**: Conceptualisation; data curation; formal analysis; investigation; methodology.

## CONFLICT OF INTEREST STATEMENT

The authors declare no conflicts of interest.

## FUNDING INFORMATION

None.

### ETHICAL STATEMENT

None.

### PEER REVIEW

The peer review history for this article is available at https://publons.com/publon/10.1002/vms3.1570.

## Supporting information


Video showing the STAPIC procedure

## Data Availability

Data are available on request.
